# Initiation of muscle protein synthesis was unrelated to simultaneously upregulated local production of IGF-1 by amino acids in non-proliferating L6 muscle cells

**DOI:** 10.1371/journal.pone.0270927

**Published:** 2022-07-08

**Authors:** Britt-Marie Iresjö, Lisa Diep, Kent Lundholm

**Affiliations:** 1 Department of Surgery, Institute of Clinical Sciences, Sahlgrenska Academy, University of Gothenburg, Gothenburg, Sweden; 2 Department of Surgery, Sahlgrenska University Hospital, Region Västra Götaland, Gothenburg, Sweden; Tohoku University, JAPAN

## Abstract

**Background:**

IGF-1 is considered an important regulator of muscle protein synthesis. However, its role in stimulation of muscle protein synthesis by amino acids (AA) is not clear, despite pronounced alterations in IGF-1 mRNA expression and signaling in muscle tissues by feeding. This study evaluates the role of locally produced IGF-1 and IGF-1 signaling when skeletal muscle protein synthesis is activated by increased amino acid availability in confluent, non-proliferating cells.

**Methods:**

L6 skeletal muscle cells were subjected to amino acid starvation (24 h, 0.14 mM) followed by 18 h amino acid refeeding in Low AA (0.28 mM) or High AA concentrations (9 mM). Protein synthesis rates were estimated by L-[U-14C]-phenylalanine incorporation into cellular proteins. IGF-1 and IGF-1 receptor mRNA expression were quantified by real time PCR. SiRNA knockdown, antibodies and chemical inhibitors were used to attenuate muscle IGF-1 production and signaling.

**Results:**

High AA concentrations (9mM) increased IGF-1 mRNA expression (+ 30%, p<0.05) and increased L-[U-14C]-phenylalanine incorporation compared to Low AA in confluent, non-proliferating muscle cells. Blocking IGF-1 signaling by chemical inhibitors reduced IGF-1 mRNA upregulation (~50%, p< 0.01), without decrease of protein synthesis. SiRNA knockdown of IGF-1 reduced protein synthesis, mainly explained by reduced cell proliferation. High AA or IGF-1 inhibitors did not change IGF-1 receptor mRNA expressions.

**Conclusion:**

Amino acids increased IGF-1 mRNA expression and stimulated muscle protein synthesis. However, simultaneous upregulation of IGF-1 mRNA did not relate to increased protein synthesis by amino acids. The results indicate that increased IGF-1 mRNA expression is rather a covariate to amino acid initiation of protein synthesis in non-proliferating muscle cells; effects that may be related to unrecognized metabolic activities, such as transport of amino acids.

## Introduction

The role of Insulin like growth factor 1 (IGF-1) for activation of skeletal muscle protein synthesis have been extensively studied, particularly in relation to exercise and muscle hypertrophy [1,2]. Our own studies have focused on IGF-1 in relationship to activation of muscle protein synthesis following feeding, with observations that mRNA levels of skeletal muscle IGF-1 and IGF-1 receptors are sensitive to alterations in oral as well as parenteral feeding [3–8]. IGF-1 mRNA levels decreased in skeletal muscles of overnight fasted mice and were rapidly restored by feeding [3–6]. Such alterations suggest that local muscle production of IGF-1 in skeletal muscles may be an activator of feeding induced muscle protein synthesis. In addition, provision of exogenous IGF-1 to overnight fasted mice increased muscle protein synthesis significantly [5,6]. However, blood levels and hepatic production of IGF-1 were unexpectedly unrelated to feeding induced skeletal muscle protein synthesis in genetically modified animals following food intake [3–5]. Thus, muscle produced IGF-1 appeared a possible important factor for muscle protein anabolism in relationship to daily feeding. However, whole body IGF-1 knockout mice exhibited feeding induced stimulation of skeletal muscle protein synthesis without alterations in muscle tissue IGF-1 mRNA levels [5]. This raised the question if muscle IGF-1 is only a covariate to amino acid activated protein synthesis in skeletal muscles.

The complexity of IGF-1 physiology and a multitude of hormone and metabolic alterations following feeding makes it difficult to clear-cut determine to what extent concomitantly increased muscle IGF-1 is necessary for activation of translation initiation of muscle protein synthesis in response to amino acids. The present study was aimed to investigate contributions of IGF-1 transcription and IGF-1 signaling in stimulation of muscle protein synthesis following increased amino acid availability.

## Material & methods

### Cultured L6 muscle cells

The rat skeletal muscle L6 cell line was used in all experiments. Our model of amino acid starvation followed by amino acid refeeding was used [9,10]. All experiments with IGF-1 antibodies or chemical inhibitors to attenuate IGF-1 signaling were performed in cells that reached confluent state before start of an amino acid starvation period. Thus, confluent cells were non-proliferating due to contact inhibition before and at the end of amino acid starvation/refeeding, as demonstrated earlier [10]. In IGF-1 knockdown experiments, using lipofectamine transfection, cells were ~80% confluent at the transfection start, since lipid-mediated transfer of short interfering RNA (siRNA) require proliferating cells for efficient uptake of siRNA. Cell cultures in siRNA transfection experiments were almost confluent at the start of the amino acid starvation period. Our confluent cell model was developed to mimic non-proliferating muscle tissue in vivo, and minimize IGF-1 effects on cell proliferation, although this could not be entirely avoided in siRNA IGF-1 knockdown experiments for obvious reasons. Briefly, confluent cells were cultured in the presence of “very low” amino acid concentrations (0.14 mM) without presence of serum and antibiotics for 24 hours. Cells were thereafter cultured in the presence of “refeeding” media (~18 hours). “Refeeding” media contained either Low (0.28mM,) or normal (9mM) concentrations of amino acids. Normal (9mM) “refeeding” media is equivalent to standard DMEM medium (Dulbecco’s modification of minimal essential medium). Nine mM represents approximately twice the amino acid concentration present in plasma after intake of a meal in healthy humans [11]. Low (0.28 mM) amino acid concentration represents a control condition earlier demonstrated to depress translation initiation [10]. Each experiment was repeated 2-7times with 2–3 independent samples per experiment as indicated in Results. A schematic timeline of the cell culture process is provided in S1 Fig.

#### Muscle protein synthesis

Protein synthesis rates were estimated by incorporation of L-[U-14C]-phenylalanine (40 μCi/μmol phe) into cellular proteins, as described [10]; rates that are equivalent to initiation of muscle protein synthesis [10]. Cells were cultured in the presence of Low- or High AA concentrations in cell culture media as described above, except for phenylalanine, which were kept at concentrations equal to Low AA media (12.5 μM) during all experimental conditions at constant specific radioactivity [10].

#### Real time PCR quantification of IGF-1 and IGF-1receptor

RNA extraction and cDNA synthesis were performed as described [9]. All RNA were of good quality with Agilent Bioanalyzer RIN values above 9.5. mRNA transcripts of IGF-1, IGF-1 receptor and Ribosomal 18s and 28s RNA gene1 (Rn1) were quantified by real time PCR on a Light-cycler 2.0 instrument. QuantiTect SYBR^®^ Green PCR reagent and pre-designed QuantiTect primer assays were purchased from Qiagen and used in all assays (Rn_Igf1_1_SG (NM_178866); Rn_Igf1r_1_SG (NM_052807); Rn_Rn1_1_SG (M11188; Referred to as 18S). Two μl (20 ng) cDNA were used in each 20 μl reaction. Real time PCR was performed with following settings; heat activation 95°C (15 min), denaturation 94°C (15 sec), annealing 55°C (20 sec), extension72°C (20 sec) for 30 or 40 cycles. Quantitative results were obtained by the relative standard curve method and results are reported as units transcript/unit Rn1 (18S) as house-keeping gene [12]. Relative Rn1 (18 S) mRNA concentrations were similar in all experimental groups. PCR efficiencies were 103, 89 and 98% for IGF-1, IGF-1r and Rn1 respectively. PCR product specificity was verified by appearance of single peaks in LightCycler melting curves and by appearance of a single band at expected base pair size estimated in Agilent Bioanalyzer. Negative and positive controls were included in each run. All samples were analyzed in duplicates.

#### IGF-1 transfection

Transfection was done using Lipofectamine RNAiMAX Transfection reagent, Opti MEM I, Silencer select negative control #2, Silencer Select GAPDH siRNA (positive control), Silencer select siRNA clone ID: s127929 (Igf-1, Gene ID Rn.201887). In amino acid-refeeding experiments, 60 000 cells/ well were seeded in 48-well plates and grown to approximately 80% confluence (DMEM with 4% glucose concentration + 10% FBS) before start of Lipofectamine transfection. Media were then changed, and Lipofectamine reagent were diluted in Opti-MEM medium according to RNAiMAX transfection protocol. Control experiments done prior to start of starvation/refeeding experiments established that most effective knockdown was achieved at 72 hours following transfection start (-77%) with 2.1 μl lipofectamine and 7.1 pmol siRNA/well. All siRNA reagents were supplied from Invitrogen-Life Technologies Europe BV.

#### Cell number

Scepter™ 2.0 Handheld Automated Cell Counter, were used to count cells before seeding in culture plates. Crystal violet staining of cell nuclei was used to estimate cell numbers at the end of siRNA transfection. Crystal violet staining were performed on plates treated similarly and in parallel with plates for protein synthesis quantification, except addition of L-[U-14C]-phenylalanine. At the end of experiments, culture media were aspirated, and cells were fixed in glacial acetic acid: 99.5% ethanol (1 part/3parts) for 15 min; thereafter air dried and then stained in 0.2% crystal violet in 20% methanol for 10 min, followed by de-staining in 20% methanol and air-dried. Finally, the cells were dissolved in 1% sodium dodecyl sulphate (SDS) and absorbance measured at 570 nm [13].

#### Inhibitition of IGF-1 signaling

Confluent L6 cell cultures were amino acid starved and refed with amino acids as described above. Inhibitors, to block IGF-1 signal transduction, were provided in cell culture refeeding media before exposure to cells. Inhibitors were provided in the following concentrations: Ly294002 (2 and 30 μM), [14]; PI-103 (100 nm and 50 μM) [15], Picropodophyllin (PPP) in 2, 10 and 20 μM [16]; and α-IR3 antibodies (anti-IGF-IR (ab-1, mouse mab) in 2 or 6 μg/ml) [17]. The lower concentrations of each inhibitor were used in a first set of experiments to measure protein synthesis rate by L-[U-14C]-phenylalanine incorporation to protein. These inhibitor concentrations were selected from literature reports [14–17]. The higher concentration of each inhibitor was determined in separate experiments; with the effective concentration of inhibitor established in experiments with addition of exogenous IGF-1 (300 ng/ml) to 9 mM amino acid refed cells. Level of inhibition was evaluated by western blot of phospho-AKT/total AKT and phospo- mTOR (not shown). The higher concentration range of inhibitors were used in experiments to measure IGF-1 and IGF-1 receptor mRNA levels. Parallel experiments of L-[U-14C]-phenylalanine incorporation were performed for some inhibitors provided at high concentrations. All inhibitors and IGF-1 were purchased from Calbiochem/Merck Millipore.

### Statistics

Results are presented as mean±SE. Statistical analyses among two or several groups were performed by ANOVA, with post-hoc comparisons using Fisher PLSD test in multi-group comparisons; p<0.05 was considered statistically significant in two-tailed tests. StatView for windows v 5.0.1 were used for statistics.

## Results

### Amino acid induced alterations of IGF-1 and IGF-1 receptor mRNA expression

**Fig 1:**
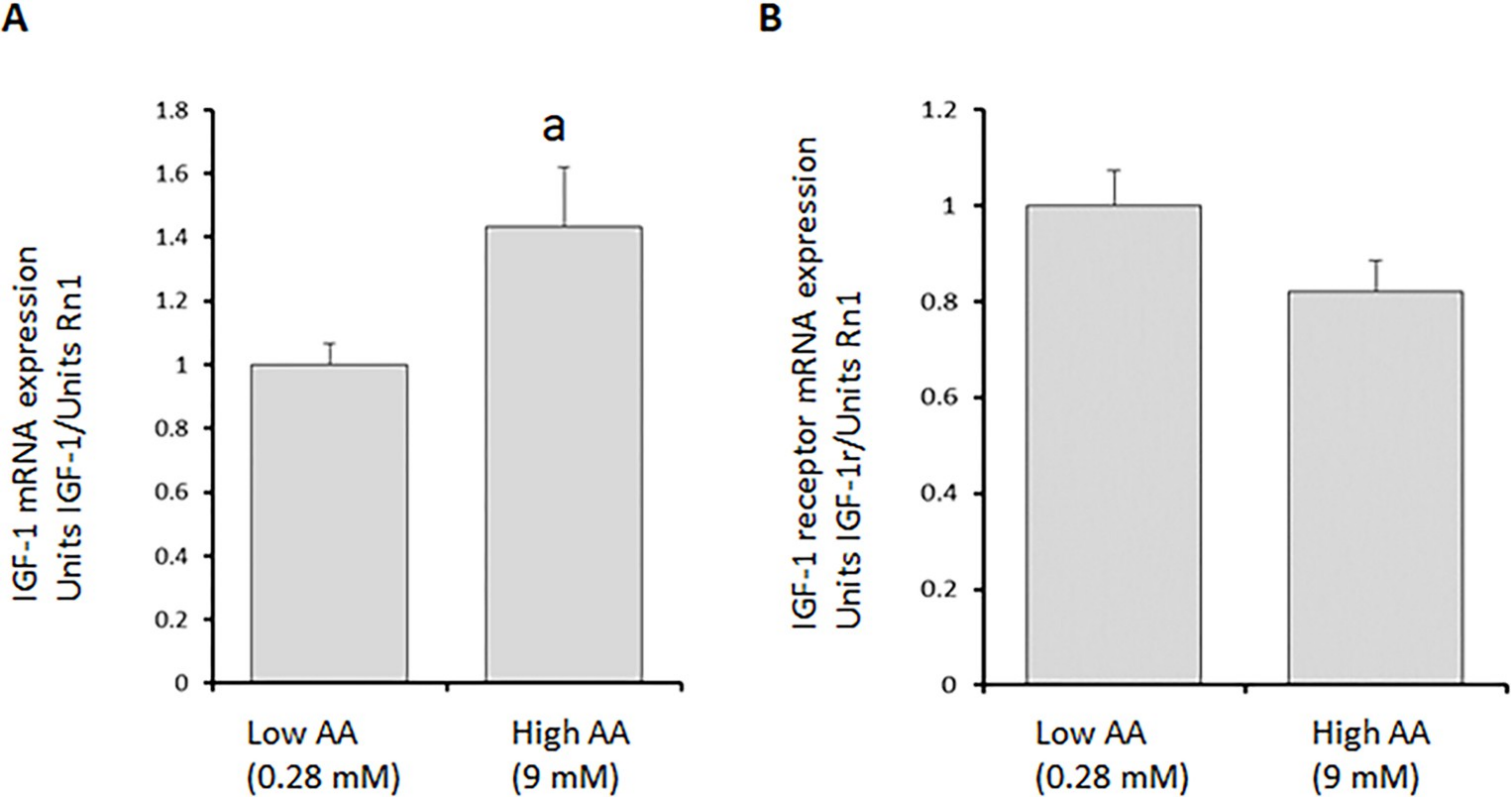
A and B. IGF-1 (**1A**) and IGF-1 receptor (**1B**) mRNA levels in Low (0.28) and High AA (9 mM) refed L6 skeletal muscle cells. IGF-1 mRNA levels increased significantly (p< 0.05), while IGF-1 receptor mRNA levels showed a decreased trend (p<0.1). Confluent, non-proliferating L6 cells were subjected to starvation in very low amino acid media for 24 hours (0.14 mM) and then cultured in presence of either Low AA (0.28 mM) or High AA (9 mM) concentrations for 18 hours, followed by RNA extraction at the end of 18hrs incubation. mRNA levels were quantified by real time PCR as described in methods. Mean±SE. a p<0.05 n = 15/group.

IGF-1 mRNA expression increased significantly in confluent, non-proliferating L6 skeletal muscle cells refed with standard AA concentration media (referred to as High AA, 9 mM), compared to cells refed with Low amino acid concentrations (Low AA, 0.28 mM), (p<0.05, n = 15) (Fig 1A). By contrast, there was a trend to reduced IGF-1-receptor expression in cells refed High AA compared to Low AA concentrations (p = 0.08, n = 15), (Fig 1B). IGF-1 and IGF-1 receptor mRNA concentrations in High amino acid refed skeletal muscle cells were similar to mRNA levels found in non-starved controls cells, kept in High amino acid media across the entire experiment period. (IGF 1, High AA RF 1.89±0.21 vs. 2.19±0.30 in non-starved controls. IGF-1 receptor, High AA 0.73±0.07 vs. 0.97±0.12 in non-starved controls; n = 7). Thus, refeeding in low amino acid concentration media (0.28 mM) was not sufficient to induce reversal of IGF-1 mRNA levels to pre-starvation levels, which refeeding in High AA media did (Fig 1A).

### IGF-1, IGF-1 receptor mRNA levels and inhibition of signal transduction

**I**nhibitors of specific steps in the IGF-1 signal transduction pathways were used to investigate contribution of IGF-1/IGF-1receptor signal transduction at amino acid induced upregulation of IGF-1 mRNA levels by amino acid refeeding. The IGF-1 signaling pathway was targeted by inhibitors of various steps in the signaling cascade (Table 1). IGF-1 receptors were blocked by either anti-IGF-1 receptor antibody (α-IR3), or chemical inhibitor PPP which inhibit IGF-1 receptor signaling by targeting Y1136 phosphorylation in the IGF-1 receptor activation loop. PI-3 kinase inhibition was obtained by either LY294002, or PI-103 which is reported to also suppress mTOR signaling. Rapamycin was used as an alternative of mTOR inhibition.

**Table 1:** IGF-1, IGF-1receptor mRNA, and L-[U-14C]-phenylalanine incorporation into proteins, in confluent non-proliferating L6 skeletal muscle cells. Cells were amino acid refed at High AA concentrations (9 mM) with or without presence of inhibitors of molecules in the IGF-1receptor signaling pathway (mean±SE, n = 4-5/group).

Condition	Inhibitor mechanism	14 C-Phenylalanine incorporation^c^	IGF-1 mRNA^d^	IGF-1 receptor mRNA^d^
High AA	-	1.00±0.01	1.00± 0.17	1.00±0.38
α-IR3 antibody 60 μg/ml	Anti IGF-1 receptor antibody	0.97±0.01	0.53±0.20 ^**b**^	1.30±0.84
PPP 20 μM	IGF-1 receptor	1.00±0.02	0.42±0.10 ^**b**^	1.12±0.56
LY294002, 20 μM	PI-3 Kinase	0.94±0.02^**a**^	0.36±0.15 ^**b**^	1.84±1.0
PI-103, 50 μM	PI-3 kinase and mTOR	nd	0.56±0.18 ^**b**^	0.98±0.42
Rapamycin	mTOR	nd	0.64±0.23 ^**b**^	0.55±0.07

a. p< 0.05 vs. High AA group (n = 5/group).

b. p< 0.01 vs. High AA group (n = 4/group).

c. DPM/vial compared to High AA refed group (DPM/mean DPM in High AA group). n = 5/group.

d. mRNA levels are expressed as units/unit 18S as described in material & methods, normalized to High AA group. n = 4/group.

nd = not determined.

IGF-1 mRNA levels were significantly lower (~30–40%) in non-proliferating cells refed with High AA media containing inhibitors, compared to control cells refed in High AA media without addition of inhibitors (p<0.01, Table 1). Thus, all IGF-1/IGF-1R signaling inhibitors blocked the High AA refeeding induced upregulation of IGF-1 mRNA, suggesting a feedback loop between intracellular IGF-1 signaling and IGF-1 mRNA transcription or translation. In contrast, IGF-1R mRNA levels were not significantly altered by any of the IGF-1 signaling inhibitors (Table 1).

### Protein synthesis estimated by L-[U-14C]-phenylalanine incorporation

**Fig 2:**
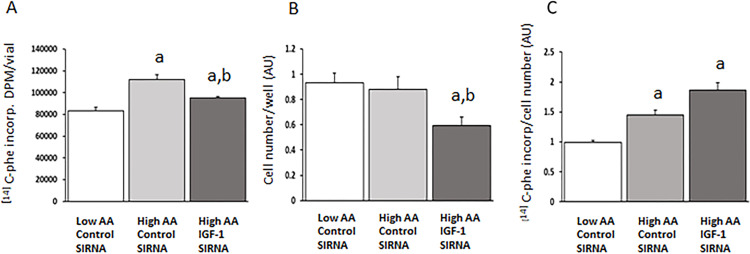
A-C. **2A.** Refeed L6 skeletal muscle cells in media with High AA concentration (9 mM) showed increased phenylalanine incorporation rates by 35% in siRNA control cells; but only 14% in IGF-1 knockdown cells compared to Low AA refed cells. **2B.** siRNA knock-down of IGF-1 reduced cell growth. Cell numbers were similar in Low AA and High AA provided cells but decreased in High AA-IGF-1 siRNA treated cells. **2C.** Differences in L-[U-14C]- phenylalanine incorporation rates between control and siRNA, IGF-1 knockdown cells did not remain accounting for cell numbers. The two High AA treated groups showed significantly increased phenylalanine incorporation compared to Low AA treated cells regardless of IGF-1 levels. Mean±SE. a p< 0.001 vs Low AA siRNA control cells. b p< 0.01 vs High AA siRNA control cells. AU. Arbitrary unit. Fold change from untreated control cells.

As expected, L-[U-14C]-phenylalanine incorporation into protein increased in non-proliferating cells refed High AA media compared to cells refed in Low AA media (~30%, p< 0.01, Fig 2A). None of the IGF-1/IGF-1R signaling inhibitors blocked the refeeding effect by High AA concentrations in the first set of experiments with IGF-1/IGF-1R signaling inhibitors provided in low concentrations (Ly294002-2 μM, PI-103-100 nm, PPP-2 and 10 μM, α-IR3 antibodies 2 μg/ml, results not shown). Such concentrations have been reported to effectively block IGF-1 mediated signaling in other cell types [14–17]. However, there was a small (~6%) but statistically significant, reduction in L-[U-14C]-phenylalanine incorporation by the PI-3 kinase inhibitor LY294002 at 20 μM concentrations (Table 1), whereas high concentrations of IGF-1R inhibitors showed no such effects in non-proliferating cells (PPP (20 μM); or α-IR3 antibody (60 μg/ml) (Table 1).

### IGF-1 knock-down by siRNA transfection

Control experiments, prior to amino acid refeeding experiments, verified effectiveness of IGF-1 knockdown to reduce IGF-1 mRNA levels (-77%). Our control experiments, in cells cultured without the amino acid starvation period and in presence of FCS, verified that IGF-1 siRNA knockdown affected protein synthesis rate. Thus, L-[U-14C]-Phenylalanine incorporation decreased by 58% in IGF-1 siRNA treated cells compared to untreated control cells (n = 8, p<0.001). However, these control experiments may to some extent be influenced by differences in cell proliferation, due to methodological requirements of lipid mediated siRNA knock-down (Material & Methods). Protein synthesis in scrambled siRNA controls (negative siRNA control cells) was similar to untreated controls (without addition of either control or IGF-1 siRNA), (93 ±2%, n = 8, p>0.05) (not shown).

We further evaluated if knockdown of IGF-1 influenced the amino acid induced increase of protein synthesis rates. High amino acid refeeding (9 mM) increased L-[U-14C]-phenylalanine incorporation by 35% in cells with normal IGF-1 mRNA expression (scrambled negative siRNA controls), while High AA refeeding in IGF-1 siRNA treated cells increased L-[U-14C]-phenylalanine incorporation by 14% only (n = 12, p<0.001; Fig 2A). However, cell number, estimated by chrystal violet staining, were significantly lower in High AA refed cells with knockdown of IGF-1 compared to High AA refed controls (scrambled negative siRNA controls) with normal mRNA IGF-1 levels (n = 8/condition; Fig 2B). There was no difference in cell number between Low AA and High AA refed cells with normal IGF-1 expression (scrambled siRNA treated, n = 8/ condition; Fig 2B). Thus, IGF-1 siRNA knockdown clearly affected cell proliferation. The lower L-[U-14C]-phenylalanine incorporation in High AA IGF-1 siRNA knockdown cells may be due to both lower number of cells and decreased protein synthesis, while incorporation at High AA, with normal IGF-1 expression, would mainly be related to increased protein synthesis in a stable pool size of cells, since no alteration in cell number occurred in these cells. The difference in phenylalanine-incorporation between control cells and IGF-1 knockdown cells disappeared after accounting for cell-number. Thus, protein synthesis rates increased similarly in High AA refed groups, regardless of IGF-1 status, when accounting for cell number (High AA IGF-1 siRNA vs. High AA Scrambled siRNA, Fig 2C n = 12). Control cells, not treated with siRNA, were included in each experiment and all conditions. There was no difference in L-[U-14C]-phenylalanine incorporation between scrambled negative siRNA control cells and control cells at any conditions (not shown).

## Discussion

Our earlier studies on patients and healthy control individuals have shown that oral and parenteral feeding are associated with elevated flux and net uptake of amino acids across whole body, arm- and leg muscle compartments [18–21]. Such findings suggest that amino acids are parts in stimulation of muscle protein synthesis at feeding; also confirmed by specific stimulation of amino acids (BCAA) in human muscle specimens in vitro [22]. Such effects, may or may not, be dependent on simultaneous effects by insulin and IGF-1 as well as other trophic factors. Therefore, in the present study, we specifically consider the contribution of IGF-1 signaling in stimulation of muscle protein synthesis following increased amino acid availability.

A large body of evidence have demonstrated that IGF-1 and downstream signaling of the IGF-1 receptor is important to stimulate skeletal muscle growth; demonstrated in various animal models with over- and under- expression of factors in the IGF1-AKT-mTOR pathway [23,24]. In addition, acute provision of recombinant IGF-1, to completely or partially starved animals, increased muscle protein synthesis [4,25,26]; and IGF-1 infusions enhanced muscle protein synthesis during hyper-aminoacidemia in healthy human subjects [27], although similar effects were not observed in post-operative patients without nutrition support [6]. In orally fed mice, administration of antibodies towards IGF-1 before feeding blunts stimulation of muscle protein synthesis [4]. Such observations suggest that IGF-1 are important in control of muscle protein synthesis also in non-growing subjects.

Our previous studies in mice with genetic deletion of hepatic IGF-1 production showed that systemic levels of IGF-1 were not critical for immediate stimulation of muscle protein synthesis at feeding [3]. Liver-IGF-1 knockouts increased muscle protein synthesis and muscle IGF-1 mRNA levels as previously demonstrated for overnight starved and refed mice [4,28]. However, it still remained unclear if elevated muscle IGF-1 production is necessary for stimulation of muscle protein synthesis following feeding? Therefore, a cell culture model was used in the present study, to distinguish between effects by amino acids or IGF-1 on stimulation of muscle protein synthesis. Our findings in the present study, show that increased protein synthesis following amino acid provision (Fig 2A), do not seem to depend on the concomitant 30% upregulation of muscle IGF-1, (Fig 1A). Also, neither blocking of IGF-1 signaling by chemical inhibitors nor knockdown of the IGF-1 gene altered stimulation of protein synthesis by amino acids. Thus, our present *in vitro* and previous *in vivo* findings [3–5] suggest that amino acids regulate IGF-1 transcription or translation, without IGF-1 being directly related to initiation of translation of muscle protein synthesis by amino acids in non-proliferating skeletal muscle cells.

The role of apparent alterations in muscle IGF-1 mRNA levels by amino acids remains elusive. A recent study reported, similar to our present study, that leucine/arginine provision increased IGF-1 secretion of cultured myocytes, without effect on gene expression of collagen related genes [29]. IGF-1 may, however, have a role to stimulate satellite cell proliferation and hypertrophy when amino acids are available at increasing levels, since satellite cells are reported sensitive to IGF-1 levels [1,24,30]. Also, our present results confirmed pronounced effects on cell proliferation by IGF-1 knockdown (Fig 2B). An extended hypothesis may be that amino acid availability regulates muscle IGF-1 production to participate in processes associated with amino acid transport [31]. Such observations are reported in other cell types, as brain cells, where inhibition of IGF-1R caused a significant decrease in glutamate transporters on cell surface [32]. Our suggestions are also supported by previous reports of altered L-type amino acid transporter levels by exogenous IGF-1 provision to skeletal muscle cell cultures [31]. Our previous *in vivo* studies confirmed a relationship among muscle IGF-1 receptors and LAT3 amino acid transporters, and showed a significant relationship by muscle IGF-1 mRNA levels and plasma leucine concentrations in parenterally fed patients [8]. Thus, IGF-1 may be related to either increased transport of amino acids to intracellular compartments, perhaps for oxidative metabolism in muscle cells; or involved in control of BCAA efflux, since LAT 3 transporters are assumed to participate in inter-organ amino acid transfer during starvation with increased release of BCAAs to the circulation [33].

Despite lack of effects on protein synthesis, several IGF-1/IGF-1R signaling inhibitors reduced the AA induced upregulation of IGF-1 mRNA levels by approximately 50% (Table 1). This indicates some kind of cross talk between IGF-1 and amino acid signaling pathways to control local IGF-1 cellular levels. Both IGF-1 and Amino acid signaling activate mechanistic target of Rapamycin (mTOR) downstream before initiation of protein synthesis but deviates in upstream regulation [34,35]. In the present study, the PI-3 kinase inhibitor (LY294002) had a small effect on protein synthesis, while IGF-1 receptor blockers lacked such effects. It is well established that IGF-1 receptor signaling depends on PI-3 kinase/Akt dependent mechanisms, although it is recently suggested that some amino acid sensing mechanisms involve generation of Ptdins3P (phosphatidylinositol 3-phosphate); and thus also depend on PI-3 kinase [36]. Therefore, it cannot be excluded that reduced phenylalanine incorporation in the presence of Ly294002 was due to inhibition of such mechanisms rather than blocked IGF-1 signaling.

In conclusion, our present study demonstrates that amino acids increased both IGF-1 mRNA expression and muscle protein synthesis. However, the significant upregulation of IGF-1 mRNA, by increased amino acid availability, was not a major factor behind induced protein synthesis in non-proliferating cells. Seen together, our present and previous results demonstrate that a primary role of IGF-1 mRNA upregulation by amino acids relates to other processes than induction of muscle protein synthesis in non-dividing skeletal muscle cells. Our findings of a clear-cut upregulation of muscle IGF-1 by amino acids may thus contribute to new understanding of factors in control of muscle protein metabolism; such as factors to maintain muscle function in progressive disease with malnutrition and flux of amino acids. Then, muscle IGF-1 may be needed to support satellite cell proliferation and other processes important to maintain pools of muscle amino acids related to cellular transporters.

## Supporting information

S1 Fig Timeline of the cell culture process with starvation/refeeding periods in IGF-1 inhibitor experiments (A) and IGF-1 knockdown experiments (B).
(TIF)
