# Supplementary figures and images for: Initiation of muscle protein synthesis was unrelated to simultaneously upregulated local production of IGF-1 by amino acids in non-proliferating L6 muscle cells
Source: PLoS One. 2022 Jul 8;17(7):e0270927. doi: 10.1371/journal.pone.0270927 (PMC9269383; doi:10.1371/journal.pone.0270927)

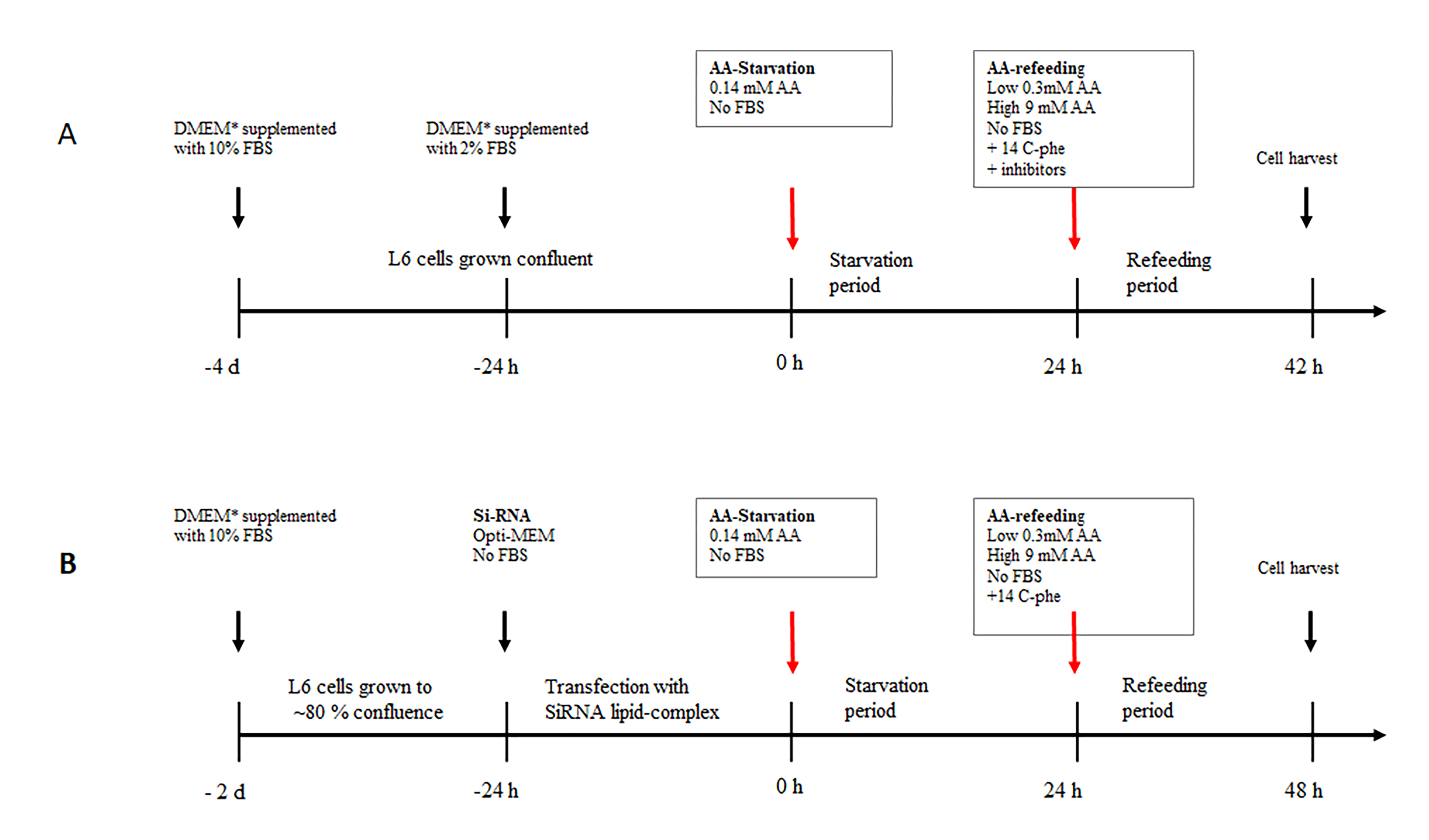

Supplement: S1 Fig — Timeline of the cell culture process with starvation/refeeding periods in IGF-1 inhibitor experiments (A) and IGF-1 knockdown experiments (B). (TIF) [file pone.0270927.s001.tif]
